# Exploring causal correlations between circulating cytokines and atopic dermatitis: a bidirectional two-sample Mendelian randomization study

**DOI:** 10.3389/fimmu.2024.1367958

**Published:** 2024-07-11

**Authors:** Zhenquan Xuan, Xuanyi Chen, Weinan Zhou, Yihang Shen, Zhe Sun, Hui Zhang, Zhirong Yao

**Affiliations:** ^1^ Dermatology Center, Xinhua Hospital, Shanghai Jiaotong University School of Medicine, Shanghai, China; ^2^ Department of Dermatology, Xinhua Hospital, Shanghai Jiaotong University School of Medicine, Shanghai, China; ^3^ Institute of Dermatology, Shanghai Jiaotong University School of Medicine, Shanghai, China

**Keywords:** atopic dermatitis, cytokines, inflammation, immunotherapy, Mendelian randomization

## Abstract

**Objectives:**

Numerous observational studies have reported associations between circulating cytokines and atopic dermatitis (AD); however, the causal relationships between them remain unclear. To explore the causal correlations and direction of causal effects between AD and levels of 91 circulating cytokines.

**Methods:**

Two-sample Mendelian randomization (MR) analyses were conducted to examine the causal relationships between 91 circulating cytokines and AD using summary statistics from genome-wide association studies (GWAS). Reverse MR analyses were performed to investigate reverse causation. Pleiotropy and heterogeneity tests were conducted to assess the robustness of the findings. Additional transcriptome database and clinical peripheral blood mononuclear cells (PBMCs) samples were utilized to validate the results of MR analyses.

**Results:**

Levels of interleukin (IL)-13, IL-18 Receptor 1, Tumor necrosis factor ligand superfamily member 14 (TNFSF14), TNF-related activation-induced cytokine (TRANCE), C-X-C motif chemokine (CXCL)11, IL-33, TNF-beta and CD5 were suggestively associated with the risk of AD (odds ratio, OR: 1.202, 95% CI: 1.018**–**1.422, *p* = 0.030; OR: 1.029, 95% CI: 1.029–1.157, *p* = 0.004; OR: 1.159, 95% CI: 1.018–1.320, *p* = 0.026; OR: 1.111, 95% CI: 1.016–1.214, *p* = 0.020; OR: 0.878, 95% CI: 0.783–0.984, *p* = 0.025; OR: 0.809, 95% CI: 0.661–0.991, *p* = 0.041; OR: 0.945, 95% CI: 0.896–0.997, *p* = 0.038; OR: 0.764, 95% CI: 0.652–0.895, *p* = 8.26e-04). In addition, levels of cytokines including Axin-1, CXCL5, CXCL10, Oncostatin-M (OSM), Sulfotransferase 1A1 (SULT1A1) and TNFSF14 were suggested to be consequences of AD (Beta: -0.080, *p* = 0.016; Beta: -0.062, *p* = 0.036; Beta: -0.066, *p* = 0.049; Beta: -0.073, *p* = 0.013; Beta: -0.089, *p* = 0.008; Beta: -0.079, *p* = 0.031). IL-13, IL-18R1, TNFSF14, and TRANCE were upregulated in both lesional skin biopsies and PBMCs from AD patients.

**Conclusion:**

The study indicates that several cytokines, including IL-13, IL-18R1, TNFSF14, TRANCE, CXCL11, IL-33, TNF-beta, and CD5, are upstream of AD development, whereas a few circulating cytokines are potentially downstream in the development of AD.

## 1 Introduction

Atopic dermatitis (AD) is the most prevalent chronic inflammatory skin disease characterized by recurrent eczematous lesions and intense pruritus (1). It affects 15–20% of children and 5–10% of adults, making it the leading cause of the global burden of skin disease (2). The pathogenesis of AD is complex and involves various aspects such as genetic predisposition, epidermal barrier dysfunction, immune dysregulation, skin microbiome abnormalities and the neuroimmune system (3). Of note, the interaction between barrier dysfunction and allergic inflammation plays a key role in the pathogenesis of AD (4). However, the etiological factors driving AD still remain controversial (5), resulting in a paucity of effective and curative therapeutic targets. Therefore, gaining a thorough mechanistic insight into pathogenesis of AD is vital and urgent.

The current controversies pertaining to the pathogenesis of AD predominantly revolve around two prevailing hypotheses. One proposed “outside-to-inside” mechanism suggests that disrupted barrier serves as the initiating factor, releasing alarmins such as interleukin (IL)-33, thymic stromal lymphopoietin (TSLP) and IL-25, which recruit subsequent immune cells and elicit T helper cell type (Th)2-skewed inflammatory responses. TSLP promotes the chemotaxis of eosinophilia and enhances the expression level of IL-4, IL-5 and IL-13 (6). Overexpressing IL-33 in keratinocytes elevated circulating levels of IL-5 and IL-13 (7). Conversely, the “inside-to-outside” proponents argue that elevated inflammatory factors disturbing permeability barrier homeostasis are the etiologies of AD (8). For example, type 2 cytokines, IL-4 and IL-13 downregulate the expression of crucial proteins, such as filaggrin, involucrin and claudin-1 in keratinocytes (9, 10). Previous observational studies found elevated alarmins and inflammatory factors in AD patients (11). However, these studies may not capture the entire picture, and establishing definite causal correlations is challenging due to potential confounding factors or reverse causation. The question of whether the inflammatory cytokines or alarmins released by keratinocytes are the cause of AD remains debatable.

Mendelian randomization (MR) is an analytical approach used to infer the causal influence of an exposure on an outcome by employing genetic variations as instrumental variables (IVs) (12). By leveraging the random allocation of alleles during meiosis, MR can help alleviate reverse causation and is less susceptible to conventional confounding variables, thereby offering more robust evidence for causal inference (13). In the year 2023, Zhao et al. embarked upon a study employing MR to elucidate the causal effects of 58 circulating proteins, each with confirmed cis-protein quantitative trait loci (pQTLs) from their investigation, on 14 immune-mediated diseases (IMDs), including AD. Their work highlighted IL-18R1 as a driving protein in the pathogenesis of AD (14). Despite these contributions, the study confronted several constraints. Methodologically, the exclusive reliance on the GSMR method potentially introduced biases, particularly in the presence of pleiotropy. Moreover, the circumscribed focus on merely 58 proteins and the implementation of unidirectional MR analyses may have obscured other crucial disease mediators and reciprocal causal influences exerted by AD on cytokine profiles. The research emphasis was skewed towards ulcerative colitis, with no independent verification of findings within AD-centric datasets, thereby leaving the comprehensive understanding of the bidirectional causal network between circulating cytokines and AD incomplete.

To address these lacunae and attain a holistic understanding of the plasma protein involvement in AD, we conducted a bidirectional two-sample MR, utilizing genome-wide association study (GWAS) summary data of 91 inflammatory cytokines to investigate the potential bidirectional causal relationship between circulating inflammatory regulators and AD. To fortify the validity of our conclusions, we have corroborated our MR findings through replication in an additional GWAS dataset. Complementary to this, we have ventured into investigating the transcriptomic signatures of the identified cytokine drivers in AD, analyzing skin biopsy specimens and peripheral blood mononuclear cells (PBMCs) sourced from AD patients. This integrative strategy serves to refine our knowledge of the complex interplay between circulating cytokines and AD, offering a more nuanced and substantiated perspective on their causal interdependencies.

## 2 Materials and methods

### 2.1 Study design

**Figure 1:**
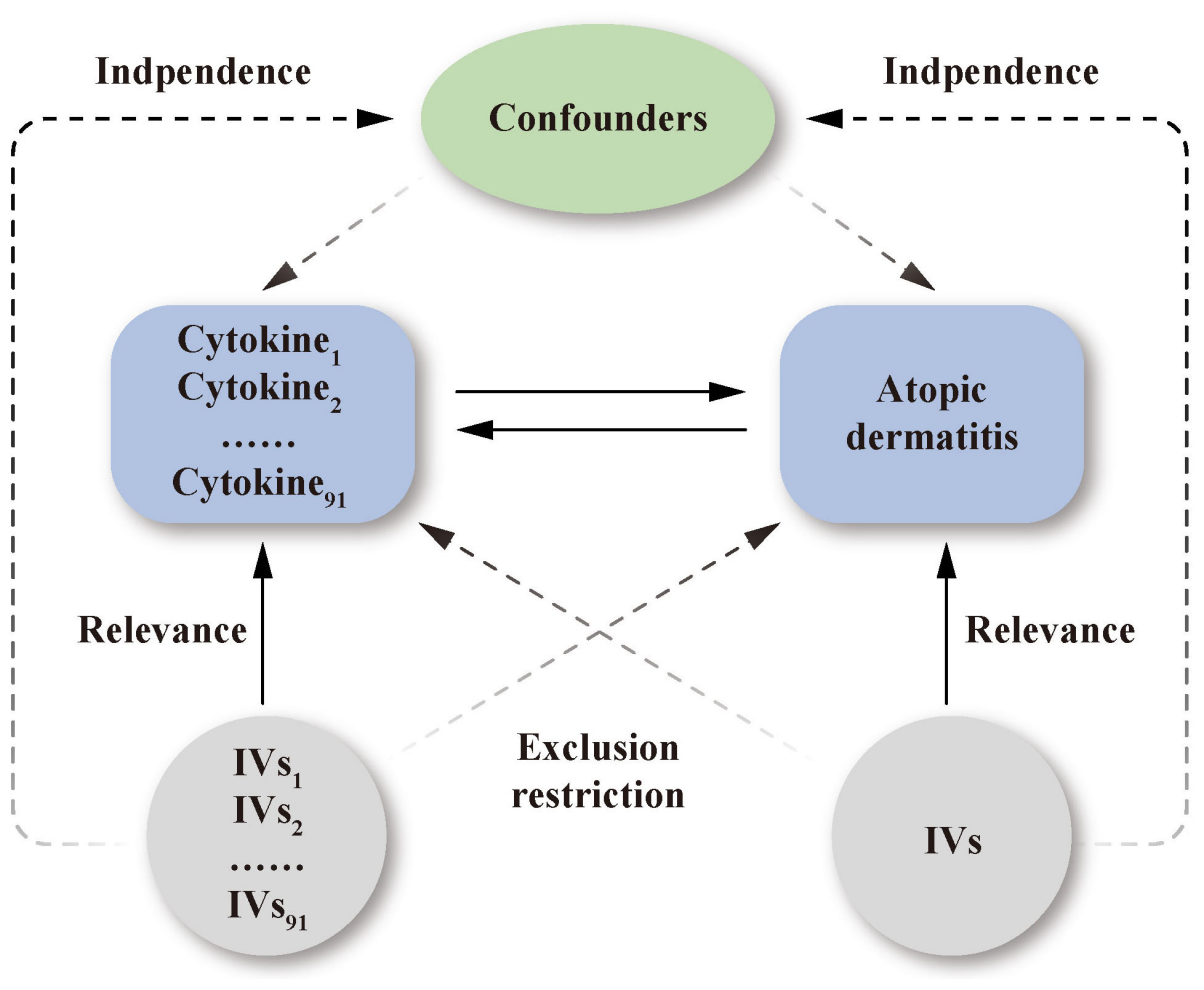
Schematic representation of the study design in this bidirectional Mendelian randomization analysis. Significant instrumental variables were selected for 91 inflammatory cytokines and atopic dermatitis. The bidirectional causal associations were then explored. Three core assumptions of MR analysis were presented in this causal directed acyclic graph: relevance, independence, and exclusion restrictions.

The study design is presented schematically in **Figure 1**. Single nucleotide polymorphisms (SNPs) were treated as valid IVs. These IVs had to fulfil three core assumptions of MR analysis: relevance, independence, and exclusion restriction (15). It is assumed that the selected IVs are associated with the risk factor (relevance), but not with any confounders in the risk factor–outcome association (independence), and that they are not linked to the outcome via any pathways other than the risk factor of interest (exclusion restriction).

### 2.2 Data sources

This MR analysis utilized two datasets derived from publicly available summarized GWAS data. The detailed data for 91 circulating cytokine levels originated from a GWAS in 14,824 healthy subjects of European ancestry, which identified multiple common genetic variants influencing circulating cytokine levels (14). The summary statistics for AD were obtained from the Early Genetics and Lifecourse Epidemiology (EAGLE) Eczema consortium’s GWAS, encompassing 20 population-based cohorts and comprising 10,788 cases and 30,047 controls of European ancestry exclusively (16). An Additional AD GWAS data for validation in this study was obtained from the FinnGen consortium R8 release data, which comprised 11,964 cases and 306,909 controls of European ancestry. There was no overlap in the selection of populations between the exposure group and the outcome group.

### 2.3 Selection of IVs

A genome-wide statistical significance threshold of < 5.0 × 10^-8^ was established to extract the SNPs robustly associated with the studied exposures. Additionally, an r^2^ threshold of 0.001 and a clump window size of 10,000 kb were set to eliminate linkage disequilibrium (17). Based on these criteria, 74 cytokines were retained for analysis. In a secondary analysis, the genome-wide significance threshold was relaxed to 5.0 × 10^-6^ to include all 91 cytokines. The strength of IVs was evaluated using the F-statistic, which is calculated as follows: F = β^2^/SE^2^, where β represents the effect size of the allele and SE represents the standard error (18). An F-statistic greater than 10 suggests the absence of weak IV bias (19).

### 2.4 Statistical analysis

Three MR analysis methods were employed, including inverse variance weighted (IVW), MR-Egger and weighted median (WM). IVW was selected as the primary method for its ability to use inverse variances of each IV as weights in calculating a valid weighted average (20). MR-Egger, on the other hand, employs a form of weighted linear regression analysis. Estimates derived from this method are robust and independent of IV validity, although they might have lower statistical precision and be susceptible to outlying genetic variations (21). The WM method mitigates estimation precision variation by assigning inverse weights based on genetic variant variance, akin to the IVW method. It shows reliability despite violated causal effects (22). While significant causal correlations were evaluated using the IVW method, the MR egger and WM methods were utilized as supplementary methods for directional validation. Odds ratios (OR) with 95% confidence intervals (CI) were reported as effect estimates for cytokines on AD. Betas with 95% CI were reported as effect estimates for AD on cytokines.

Pleiotropy was assessed using MR-Egger regression. Additionally, the presence of pleiotropy and identification of outlying SNPs were determined through the MR-Pleiotropy RESidual Sum and Outlier (PRESSO) test (23). Heterogeneity was assessed using Cochran’s Q test, with a *p*-value < 0.05 indicating its presence.

When analyzing multiple outcomes, significance thresholds were adjusted using the Bonferroni method. Statistical significance was indicated by a *p*-value lower than the Bonferroni-corrected threshold. Correlations with *p*-values less than 0.05, but not reaching the Bonferroni-corrected significance level, were considered suggestively significant. All analyses were performed using R version 4.2.2, with the software packages “TwoSampleMR” and “MRPRESSO”.

### 2.5 Transcriptome analysis

Data of transcriptome profiles were extracted from Gene Expression Omnibus (GEO) accession no. GSE157194 consisting of 166 high-throughput RNA-sequencing expression profiles of lesional and non-lesional skin biopsies collected from 57 AD patients (24). Data cleaning and statistical analysis were applied with GEO2R software (25), and the comparison of gene expression between the lesional and non-lesional samples was applied with T-test.

### 2.6 Validation of the results in the PBMCs of AD patients

Blood samples (5 mL) were collected from hospitalized AD patients and healthy individuals undergoing physical examination at Xinhua Hospital, Shanghai Jiaotong University School of Medicine, anticoagulated with ethylenediamine tetraacetic acid, and sent to the laboratory within 2 h. The blood samples were centrifuged at 200 g/min 4°C for 5 min, and then the sediment was separated for preparing PBMC by standard Ficoll-Hypaque density centrifugation. In accordance with standard procedures, RNA was extracted from PBMCs and subjected to quality control and reverse transcription. The resulting cDNA was then amplified using specific primers for RT-qPCR. The internal reference gene *GAPDH* was used to calculate the relative expression levels of the target genes, based on the Ct values obtained from the RT-qPCR reaction. The relative mRNA expression was calculated based on the 2^−ΔΔCt^ approach. Approval for the current study was granted by the Ethics Committee of Xinhua Hospital, Shanghai Jiaotong University School of Medicine. The primer sequences are listed in **Table 1**. The clinical sample information is presented in **Supplementary Table S1**.

**Table 1:** The primers used in this study.

Gene name	Primer	Sequence (5’-3’)	Length (bp)
*GAPDH*	Forward	5’- TCAAGAAGGTGGTGAAGCAGG-3’	115 bp
	Reverse	5’-TCAAAGGTGGAGGAGTGGGT-3’	
*IL18R1*	Forward	5’-ACACTGGTCAACAGCACATCA-3’	90 bp
	Reverse	5’-CTCGGCGTTCTTCTTTATCGT-3’	
*TNFSF14*	Forward	5’-GGAAATGCTTGCTGATTGA-3’	107 bp
	Reverse	5’-TTGTGAAAG AGT GGC TGA GAT-3’	
*TRANCE*	Forward	5’-CGGTACACGACTCAGTATCCA-3’	77 bp
	Reverse	5’-AGTCTAACATCTCCCACTGGC-3’	
*IL13*	Forward	5’-ATGAGTGTGTTTGTCACCGT-3’	79 bp
	Reverse	5’-CCTGAGTCTCTGAACCCTTG-3’	

## 3 Results

### 3.1 Causal effects of circulating cytokines on AD

**Figure 2:**
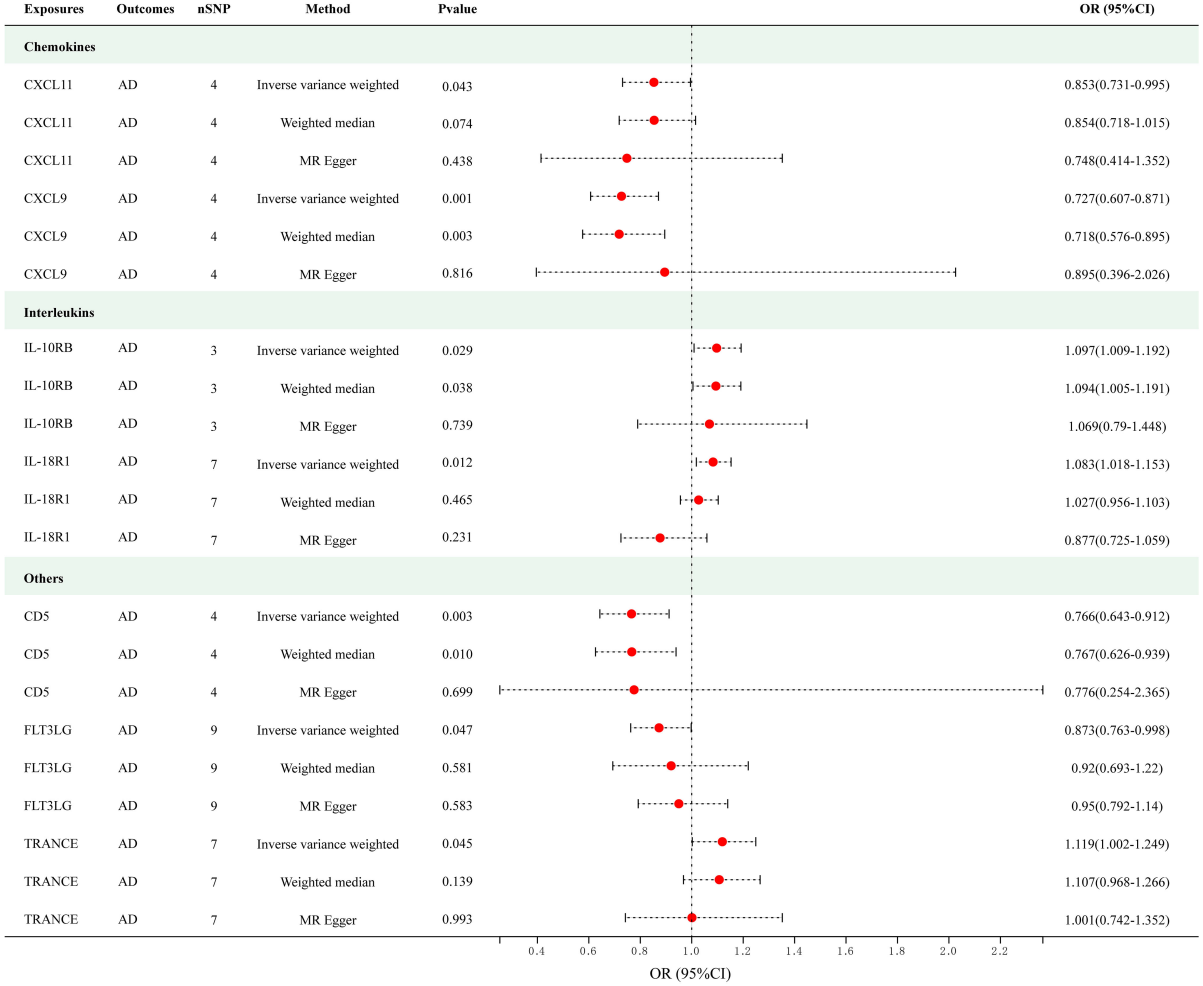
Causal correlations of 7 cytokines with atopic dermatitis (SNP associations reaching a significance level of *p* < 5×10^-8^). CXCL11, C-X-C motif chemokine 11; CXCL9, C-X-C motif chemokine 9; IL-10RB, interleukin-10 receptor subunit beta; IL-18R1, interleukin-18 receptor 1; CD5, T-cell surface glycoprotein CD5; FLT3LG, Fms-related tyrosine kinase 3 ligand; TRANCE, TNF-related activation-induced cytokine.

**Figure 2** presents the outcomes of the MR analysis on the causal relationship between circulating cytokines and AD at a significance threshold of < 5.0 × 10^-8^. The findings of the IVW method revealed that IL-10 receptor subunit beta (IL-10RB) (OR = 1.097, 95% CI = 1.009–1.192, *p* = 0.029), IL-18 receptor 1 (IL-18R1) (OR = 1.083, 95% CI = 1.018–1.153, *p* = 0.012) and TNF-related activation-induced cytokine (TRANCE) (OR = 1.119, 95% CI = 1.002–1.249, *p* = 0.045) were correlated with an elevated risk of AD. In addition, the IVW method showed T-cell surface glycoprotein CD5 (CD5) (OR = 0.766, 95% CI = 0.643–0.912, *p* = 0.003), C-X-C motif chemokine (CXCL) 11 (OR = 0.853, 95% CI = 0.731–0.995, *p* = 0.043), CXCL9 (OR = 0.727, 95% CI = 0.607–0.871, *p* = 5.35e-04) and Fms-related tyrosine kinase 3 ligand (FLT3LG) (OR = 0.873, 95% CI = 0.763–0.998, *p* = 0.047) were associated with the decreased risk of AD. Among these cytokines, CXCL9 met the Bonferroni-corrected threshold range (*p* = 0.05/51 = 9.80e-04), whereas the others did not. The scatter plots of MR analyses for above 7 cytokines are shown in **Supplementary Figure S1**. The specific SNP profiles, the pleiotropy and heterogeneity results of these 7 groups, are presented in **Supplementary Tables S3** and **S4**. No evidence of heterogeneity and pleiotropy was observed (*p* > 0.05), and all F-statistics for the groups were >10, indicating the absence of weak IV bias. No outliers were found. **Supplementary Table S5** presents the MR analyses involving 51 cytokines and AD.

**Figure 3:**
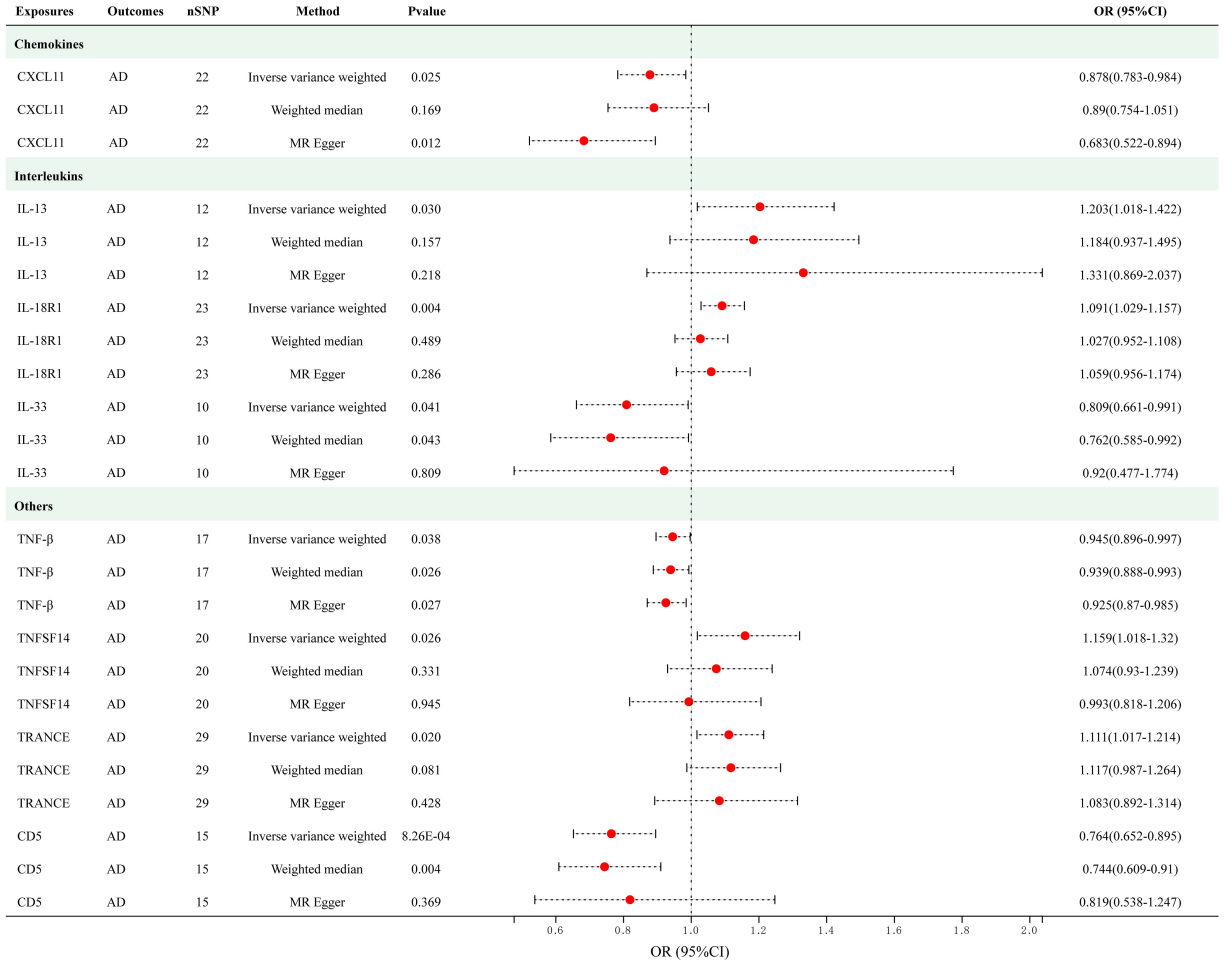
Causal correlations of 8 cytokines with atopic dermatitis (SNP associations reaching a significance level of *p* < 5×10^-6^). CXCL11, C-X-C motif chemokine 11; IL-13, interleukin-13; IL-18 R1, interleukin-18 receptor 1; IL-33, interleukin-33; TNF-β, TNF-beta; TNFSF14, Tumor necrosis factor ligand superfamily member 14; TRANCE, TNF-related activation-induced cytokine; CD5, T-cell surface glycoprotein CD5.

As presented in **Figure 3**, employing a significance threshold of <5.0 × 10^-6^, 8 sets of suggestively significant correlations (*p* < 0.05) were obtained. The findings of the IVW method revealed that elevated levels of IL-13 (OR = 1.202, 95% CI = 1.018–1.422, *p* = 0.030), IL-18R1 (OR = 1.029, 95% CI = 1.029–1.157, *p* = 0.004), tumor necrosis factor ligand superfamily member 14 (TNFSF14) (OR = 1.159, 95% CI = 1.018–1.320, *p* = 0.026) and TNF-related activation-induced cytokine (TRANCE) (OR = 1.111, 95% CI = 1.016–1.214, *p* = 0.020) were suggestively correlated with an increased risk of AD. In addition, the IVW method also revealed causal relationships between circulating levels of CXCL11 (OR = 0.878, 95% CI = 0.783–0.984, *p* = 0.025), IL-33 (OR = 0.809, 95% CI = 0.661–0.991, *p* = 0.041), TNF-beta (TNF-β) (OR = 0.945, 95% CI = 0.896–0.997, *p* = 0.038) and CD5 (OR = 0.764, 95% CI = 0.652–0.895, *p* = 8.26e-04) and a decreased risk of AD. After applying Bonferroni correction (*p* = 0.05/91 = 5.50e-04), none of the cytokines showed a statistically significant association with AD. The scatter plots of MR analyses for above 8 cytokines are shown in **Supplementary Figure S2**. Detailed information on the SNP profiles, pleiotropy, and heterogeneity are presented in the tables in the **Supplementary Tables S6** and **S7**. No heterogeneity or pleiotropy was observed in the cytokines, and there was no evidence of weak IV bias. No outliers were found. The details of MR analyses involving 91 cytokines and AD are presented in **Supplementary Table S8**.

### 3.2 Causal effects of AD on circulating cytokines

**Figure 4:**
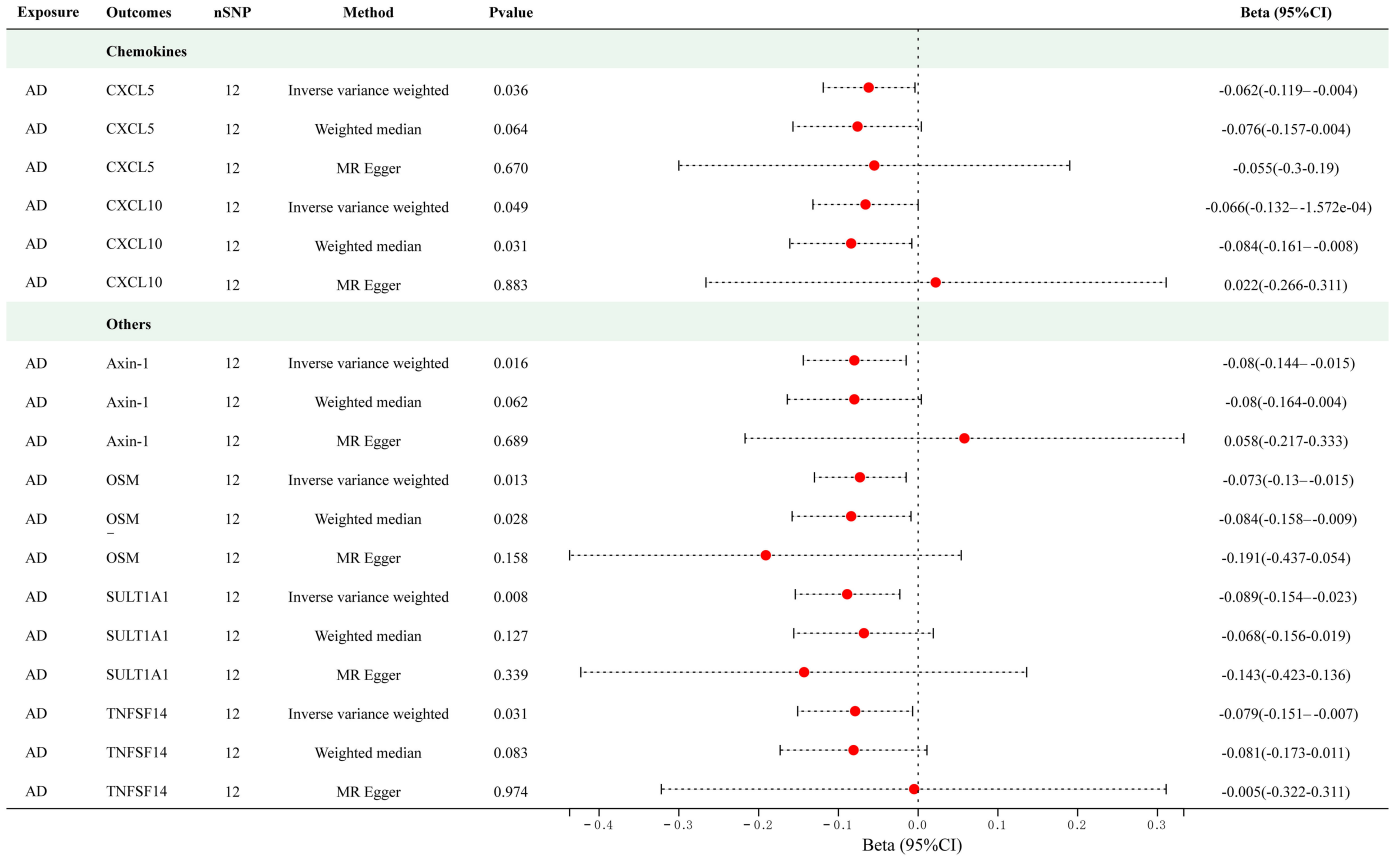
Causal correlations of atopic dermatitis with 6 cytokines (SNP associations reaching a significance level of *p* < 5×10^-8^). CXCL5, C-X-C motif chemokine 5; CXCL10, C-X-C motif chemokine 10; OSM, Oncostatin-M; SULT1A1, Sulfotransferase 1A1; TNFSF14, Tumor necrosis factor ligand superfamily member 14.

Twelve significant SNPs were extracted as the IVs for AD. **Figure 4** presents the results of the MR analysis investigating the causality between AD and inflammatory cytokines. The findings of the IVW method revealed that AD was suggestively associated with an decreased level of Axin-1 (Beta = -0.080, 95% CI = -0.144–0.015, *p* = 0.016), CXCL5 (Beta = -0.062, 95% CI = -0.119–0.004, *p* = 0.036), CXCL10 (Beta = -0.062, 95% CI = -0.132–1.572e-04, *p* = 0.016), Oncostatin-M (OSM) (Beta = -0.073, 95% CI = -0.13–0.015, *p* = 0.013), Sulfotransferase 1A1 (SULT1A1) (Beta = -0.089, 95% CI = -0.154–0.023, *p* = 0.008) and TNFSF14 (Beta = -0.079, 95% CI = -0.151–0.007, *p* = 0.031). The scatter plots of MR analyses for above results are shown in **Supplementary Figure S3**. **Supplementary Tables S9** and **S10** include information about the SNPs, heterogeneity, and pleiotropy for these groups. No heterogeneity or pleiotropy was observed, and the F-statistics indicated the absence of weak IV bias. No outliers were found. Detailed results of reverse MR analyses are presented in **Supplementary Table S11**.

### 3.3 Validation of the results in FinnGen GWAS data

To further verify our results, we utilized an additional AD GWAS dataset from the FinnGen consortium R8 release data, which comprised 11,964 cases and 306,909 controls of European ancestry. As presented in **Supplementary Figure S4**, employing the IVW methodology, we observed a reduced risk of AD associated with CD5 (OR = 0.860, 95% CI = 0.778–0.952, *p* = 0.004). Furthermore, the IVW analysis revealed an elevated AD risk correlated with IL-18R1 (OR = 1.103, 95% CI = 1.048–1.161, *p* = 0.046). While the IVW method did not yield uniformly conclusive results, alternative methodologies, specifically the MR Egger (OR = 1.160, 95% CI = 1.021–1.317, *p* = 0.034) and the WM method (OR = 1.183, 95% CI = 1.051–1.332, *p* = 0.005), suggested potential correlations implicating TNFSF14 with a heightened risk of AD. Also, the MR Egger analysis reinforced an augmented AD risk in connection with TRANCE (OR = 1.139, CI =1.011–1.283, *p* = 0.044). The scatter plots of MR analyses for above results are shown in **Supplementary Figure S5**. Detailed results of the MR analyses for validation are presented in **Supplementary Table 12**. No heterogeneity or pleiotropy was observed, and no outliers were found. Collectively, these outcomes fortify the probable causative roles of CD5, IL-18R1, TNFSF14, and TRANCE in AD etiology, significantly bolstering the rigor and reproducibility of our initial discoveries to a considerable extent.

### 3.4 Expression patterns of protein drivers in AD patients

**Figure 5:**
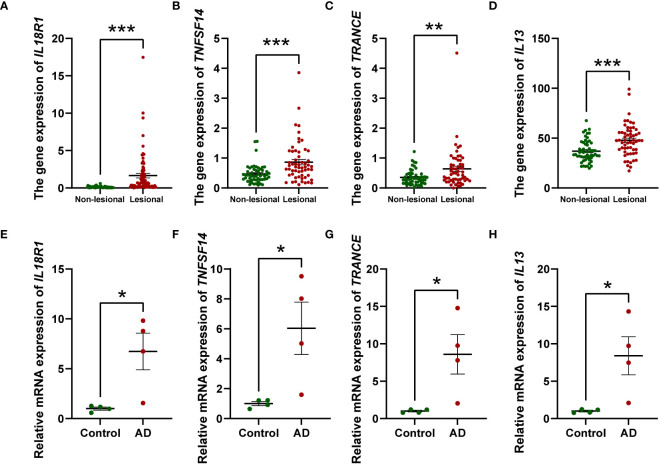
Relative mRNA levels of *IL18R1*, *TNFSF14*, *TRANCE*, and *IL13* in AD lesional samples compared to non-lesional samples **(A–D)** and PBMCs from AD patients compared to healthy individuals (n = 4) **(E–H)** investigated by RT-qPCR. The relative mRNA expression was calculated based on the 2^−ΔΔCt^ approach. Data are presented as mean ± SEM of three independent experiments. The significance level was set at *p* ≤ 0.05 (*), *p* ≤ 0.01 (**), and *p* ≤ 0.001 (***).

To gain more mechanistic insights into AD, we undertook a more in-depth investigation into four inflammatory factors that have emerged as elevating risks for AD, namely IL-13, IL-18R1, TNFSF14, and TRACNE. Considering that circulating inflammatory factors primarily originate from tissues and cellular components within the circulation, we sought to investigate the expression profiles of these four factors in both skin tissue and PBMCs to further validate our research outcomes. To this end, we compared the expression levels of these factors in lesional and non-lesional skin biopsies from 57 AD patients utilizing transcriptome databases. We observed that the gene expression of *IL18R1* (**Figure 5A**), *TNFSF14* (**Figure 5B**), *TRANCE* (**Figure 5C**) and *IL13* (**Figure 5D**) was significantly increased in AD lesional samples compared with non-lesional samples, which is consistent with the results of our MR analysis. Augmenting these bioinformatic analyses, we executed qPCR assays on PBMCs derived from AD patients and healthy volunteers to contrast the expression patterns of the four inflammatory markers. Our results showed that all of them were elevated in PBMCs from AD patients compared to healthy individuals (**Figures 5E–H**). These combined approaches serve to robustly corroborate and fortify the conclusions drawn from the MR study.

## 4 Discussion

AD is a systemic inflammatory disease characterized by variational levels of inflammatory cytokines in the serum of affected individuals (26). The roles of circulating inflammatory cytokines in AD, however, are subject to ongoing debate. It remains unclear whether they serve as primary drivers of AD or if they are instead downstream players during progression of the disease. The “outside-to-inside” mechanism suggests that the dysfunction of the barrier is the primary etiology of AD and subsequently induces immunological alterations (27). On the contrary, the “inside-to-outside” proposal insists inflammatory factors serve as initial pathological causes to disturb permeability barrier homeostasis. To advance our understanding, there is an imperative need for meticulous studies, such as MR studies to furnish empirical evidence supporting either hypothesis.

Until now, several studies have explored the casual effects of circulating cytokines and skin diseases utilizing MR. Li et al. investigated the causal relationship between inflammatory cytokines and three immunoinflammatory dermatoses, and revealed inhibitory roles of IL-4 and IL-1RA in the risk of developing AD (28). The findings of this study diverge from ours, potentially attributable to variations in GWAS selections and dissimilarities in study designs. This unidirectional MR study confined its scope to an examination of only 41 inflammatory mediators and did not validate the results in clinical samples, thereby imparting a considerable degree of limitation to the conclusions drawn. Another work featured an extensive pQTL analysis employing the Olink Target platform, alongside MR investigations utilizing 58 plasma proteins with cis-pQTLs, to clarify the causal roles of plasma proteins in AD (14). And their recognition of IL-18R1 as a driver of AD resonates with our findings. Despite the breadth of this investigation, its analytical limitations and inadequate focus on AD might hindered the dissection of the intricate causal dynamics between inflammatory cytokines and AD. To unravel the complex etiological mechanisms of AD, we capitalized on the GWAS data encompassing 91 plasma cytokines from the original study, conducting a bidirectional two-sample MR analysis to scrutinize causal links between these cytokines and AD. To surmount prior methodological limitations, we adopted a trio of MR approaches – IVW, MR-Egger, and WM – to mitigate biases effectively. To cover all 91 cytokines, we set a genome-wide significance threshold of <10^-6^ when discussing the results. When considering circulating cytokines as the exposures, our analysis showed that IL-13, IL-18R1, TNFSF14 and TRANCE were potential upstream causal contributors to the development of AD, while CXCL11, IL-33, TNF-β and CD5 appeared to be associated with the decreased risk of AD. Additionally, when using AD as the exposure in our MR analysis, we found that AD was associated with reduced circulating levels of Axin-1, CXCL5, CXCL10, OSM, SULT1A1 and TNFSF14 through causative mechanisms. To validate these findings, we capitalized on additional GWAS data from the FinnGen biobank, reinforcing the significant causal relationships between AD and CD5, IL-18R1, TNFSF14, and TRANCE. Our investigation further disclosed elevated expression of four driver cytokines in lesional versus non-lesional skin biopsies from AD patients. Complementary RT-qPCR assays in PBMCs from AD patients demonstrated elevated expression of these cytokines relative to healthy controls, consolidating the validity of our research conclusions through an integrated strategy.

Extensive research has explored the correlations between inflammatory factors and AD, which supported the results of our study. IL-13, predominantly secreted by Th2 cells and group 2 innate lymphoid cells (ILC2s) in the dermis, has emerged as a cardinal type 2 cytokine driving inflammatory processes in AD (29). Its pathogenic role encompasses a broad spectrum of effects on cutaneous biology, notably the recruitment of inflammatory cells, modulation of the skin microbiome, and compromise of the epidermal barrier (29). IL-18, initially characterized as an “interferon-γ-inducing factor”, is synthesized by a diverse array of cell types, inclusive of keratinocytes, dermal macrophages, and dendritic cells (30). The level of IL-18 in AD lesions was significantly higher in comparison to healthy controls and was correlated with AD severity (31). The binding of IL-18 to its receptor IL-18R1 on mast cells and basophils amplifies the concentrations of IL-4 and IL-13 within AD lesions, culminating in inflammatory cascades (32). TNFSF14, chiefly synthesized by CD4^+^ and CD8^+^ T cells, has garnered attention in the context of AD (33). Compared to healthy counterparts, AD patients exhibit markedly elevated serum levels of TNFSF14, positioning it as a potential biomarker for assessing disease severity (34). The role of TNFSF14 in AD is multifaceted, with implications for keratinocyte stimulation. Conditional ablation of TNFSF14 receptors in keratinocytes has been shown to confer protection against allergen-induced skin inflammation in murine models of AD. Furthermore, exposure of human keratinocytes to recombinant TNFSF14 triggers transcriptional alterations in genes pertinent to AD pathogenesis (35). Our study suggested causal associations between these cytokines and AD, which further substantiates their roles as upstream factors in the pathogenesis of AD. Limited studies have investigated the role of TRANCE in the pathogenesis of AD. TRANCE, a member of the TNF superfamily, is mainly expressed by stromal cells, activated T cells and B cells (36, 37). Our MR analysis found that elevated levels of circulating TRANCE were associated with an increased risk of AD, providing new mechanistic insights into the pathogenesis of AD. The TRANCE-involved pathways have been reported to enhance the secretion of inflammatory cytokines in dendritic cells (38), and the characteristic expansion of inflammatory dendritic cells in AD lesions has been substantiated through several single-cell RNA sequencing analyses (39, 40). Therefore, we hypothesize that TRANCE may instigate AD by priming dendritic cells to adopt a pro-inflammatory phenotype.

Acknowledging the significance of targeting pivotal upstream cytokines in the management of AD, recent advancements have indeed underscored their therapeutic potential. Notably, the approval of dupilumab, an antagonist of IL-4 and IL-13, for treating patients with moderate-to-severe AD exemplifies this promising approach (41). Our research findings bear considerable therapeutic implications, as they unveil novel targets that may permit targeted intervention like biologicals on the very origin of AD pathology. Rigorous validation and well-designed clinical trials will be essential in ascertaining the therapeutic efficacy and safety of targeting these newly identified cytokines in patient populations.

In addition, our MR analysis revealed negative causal associations between several circulating cytokines and the risk of AD, including TNF-β, CD5, CXCL11, and IL-33. Previous research has explored the roles of these cytokines. For instance, it has been reported that TNF-β downregulates the expression of IL-5 and IL-13 in PBMCs, and the blood levels of TNF-β were significantly lower in AD patients compared to normal levels (42), which aligns with our results. In addition, TNF-β is required for efficient regulatory T cells (Tregs) migration and suppressive function (43, 44), which might be helpful in treating AD as Tregs are essential in controlling type 2 inflammation (45). Although the down-regulatory roles of some factors in inflammatory responses were well-established, their associations with AD have received little attention. CD5, a transmembrane receptor (46), has been identified as a negative regulator of the T-cell receptor signaling pathway in both thymocytes and T lymphocytes (47–49). CD5 deficiency leads to an enhanced phosphorylation of Signal Transducer and Activator of Transcription 3 (STAT3) in T lymphocytes and primes them for an increased production of Interleukin-17A (50), a cytokine that has been substantiated as pivotal in the pathogenesis of AD, particularly in its more severe manifestations (51). Furthermore, intravenous immune globulin (IVIG) therapy increased the serum CD5 levels in patients with AD, along with the improvement of disease severity (52). The chemokine CXCL11 binds to CXCR3 receptors on CD4^+^ T cells (53) and inhibits inflammatory autoimmune responses (54). Further investigation is required to explore associations between these cytokines and AD. Notably, we found that higher levels of IL-33 genetic prediction were associated with a lower risk of atopic dermatitis, which seemingly contradicts prior research findings. As an alarmin, IL-33 recruits various cells to the dermis, including ILC2, which produce type 2 cytokines like IL-5 and IL-13 (55, 56). However, the primary site where IL-33 functions as an alarmin is predominantly limited to the skin, rather than the bloodstream. In addition, it is noteworthy that IL-33 antibody failed to demonstrate any benefits in a Phase II double-blind randomized controlled study involving patients with moderate-to-severe AD (57). Therefore, the role of circulating IL-33 in AD patients may be less prominent than initially hypothesized, and its precise function within the disease context remains an intriguing question awaiting further exploration.

In clinical practice, the restoration of immune cell populations and functionality through cytokine therapy presents a promising avenue for the treatment of inflammatory diseases. A case in point is the utilization of low-dose IL-2 therapy, given its pivotal role in expanding the Treg population (58). This approach has been successfully employed in the management of various inflammatory diseases, notably systemic lupus erythematosus and rheumatoid arthritis (59). Our research findings provide the potential for employing low-dose injection of these key cytokines as a therapeutic strategy for AD, and further foundational and clinical investigations are expected to substantiate the efficacy and ascertain the safety of such an intervention.

When AD was defined as the exposure in the MR analysis, it suggestively resulted in decreased circulating levels of Axin-1, CXCL5, CXCL10, OSM, SULT1A1 and TNFSF14 through causative pathways. The roles of several aforementioned cytokines in AD are sparingly investigated. For example, current research investigating Axin-1, a scaffold protein, and SULT1A1, an essential isoform of phenol SULT enzymes, is mainly focused on their roles in oncogenesis in different tissue (60–63). Further clinical and basic studies are imperative to validate our findings. In contrast, some of these inflammatory factors are tightly linked with AD, according to previous studies. OSM, a pro-inflammatory cytokine released by macrophages, monocytes, dendritic cells, and T lymphocytes (64), is up-regulated in the AD lesions (65). OSM can sensitize sensory neurons during episodes of inflammatory pruritus (66). CXCL5, a neutrophil-attracting chemokine, and CXCL10, a member of Th1 chemokine, are significantly up-regulated in skin biopsies of AD patients (67, 68). Of note, our results revealed that AD can decrease the serum levels of TNFSF14 in a causal pathway, while in turn, TNFSF14 is an etiology of AD according to our study when it is considered as an exposure. It is possible that AD reduces the levels of these cytokines through a negative feedback mechanism. Additional research is necessary to explore their underlying mechanistic roles in AD.

Our findings seem to align with the “inside-to-outside” hypothesis as several inflammatory cytokines have been implicated as etiological factors in AD, whereas none of the alarmins show a causal association with AD. However, our study has several limitations. First, cytokines analyzed in this study were quantified in plasma rather than in skin tissue. Proteomic analyses revealed that increases in inflammatory proteins in AD-affected skin compared to healthy controls were more pronounced than those found in the blood, suggesting that the skin exhibits the most evident inflammatory profile in AD (69). Therefore, circulating inflammatory factors may not accurately reflect the inflammatory status of the skin tissue, and a sole focus on inflammatory factors in the blood may neglect other crucial factors and fail to provide a comprehensive understanding of AD. Currently, there is relatively limited GWAS data on inflammatory factors in skin tissue, making it challenging to infer causal relationships between cutaneous inflammatory factors and AD using MR. Second, our findings may primarily apply to European populations due to the origin of the dataset, failing to capture the whole picture. Recent research indicated that AD exhibits distinct pathological mechanisms among various ethnicities and age groups (70). Th2 and Th17 immune pathways has been reportedly activated in Asian patients, while patients of European descent with atopic dermatitis exhibit predominantly Th2 pathway activation (71). In the future, more heterogeneous GWAS data, or those specifically stratified by age or disease severity in AD patients may be utilized as better sources for such MR analysis to identify more precise causal relationships between inflammatory factors and AD. Finally, since cytokine levels as dynamic biomarkers are subject to fluctuations, our analysis failed to capture the temporal variability of cytokines. Therefore, although the causative roles of circulating cytokines in AD support the “inside-to-outside” proposal, the “outside-to-inside” theory cannot be dismissed. Further biological experiments and clinical trials are expected to verify these hypotheses and provide more specific mechanistic insights.

## 5 Conclusion

**Figure 6:**
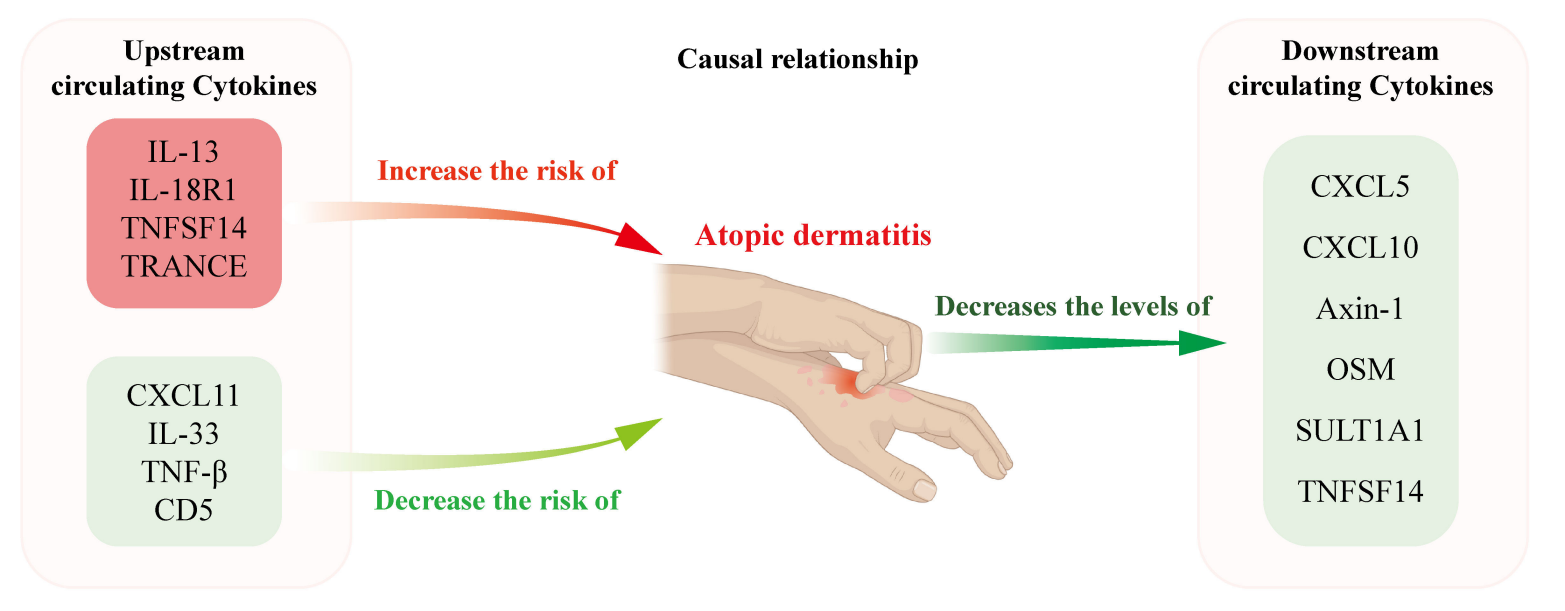
Schematic representation illustrating the postulated causal relationships and direction of causal effects between AD and the levels of circulating cytokines. Elevated circulating levels of IL-13, IL-18R1, TNFSF14, and TRACNE were suggestively associated with an increased risk of AD, whereas TNF-β, CD5, CXCL11, and IL-33 levels were negatively associated with the risk of AD. In contrast, AD was associated with reduced circulating levels of Axin-1, CXCL5, CXCL10, OSM, SULT1A1 and TNFSF14.

**Figure 6** depicts the findings of our study, which implicates elevated serum concentrations of IL-13, IL-18R1, TNFSF14 and TRANCE as being suggestive of an elevated risk for AD. This associative link is corroborated by observations of elevated gene expression levels in both lesional skin tissues and PBMCs derived from AD patients, whereas TNF-β, CD5, CXCL11 and IL-33 levels were negatively associated with the risk of AD. In contrast, AD was associated with reduced circulating levels of Axin-1, CXCL5, CXCL10, OSM, SULT1A1 and TNFSF14. Our research findings carry considerable therapeutic potential, as they unveil novel targets that may permit targeted intervention like biologicals on the very origin of AD pathology. Also, these discoveries herald the prospect of employing injections of immunoregulatory cytokines as a promising and efficacious therapeutic strategy. By identifying these critical junctures in the disease’s progression, we lay the groundwork for treatments that modulate the body’s intrinsic cytokine network, potentially leading to more potent and tailored interventions for AD patients.

## Data availability statement

Publicly available datasets were analyzed in this study. This data can be found here: The data that support this study are openly available. GWAS data of AD for exploration could be downloaded from ieu open gwas project (https://gwas.mrcieu.ac.uk/datasets/ieu-a-996). GWAS data of AD for validation could be downloaded from the FinnGen consortium R8 release data (https://console.cloud.google.com/storage/browser/finngen-public-data-r8/summary_stats). Summary statistics of 91 circulating cytokines could be downloaded from GWAS Catalog (https://www.ebi.ac.uk/gwas/publications/37563310).

## Ethics statement

The studies involving humans were approved by Xinhua Hospital, Shanghai Jiaotong University School of Medicine. The studies were conducted in accordance with the local legislation and institutional requirements. The participants provided their written informed consent to participate in this study.

## Author contributions

ZX: Conceptualization, Data curation, Supervision, Writing – original draft. XC: Methodology, Supervision, Writing – original draft. WZ: Data curation, Methodology, Writing – original draft. YS: Data curation, Formal Analysis, Writing – original draft. ZS: Formal Analysis, Writing – original draft. HZ: Writing – original draft, Writing – review & editing. ZY: Supervision, Writing – review & editing.
