# Supplementary material for: Exploring causal correlations between circulating cytokines and atopic dermatitis: a bidirectional two-sample Mendelian randomization study
Source: Front Immunol. 2024 Jul 11;15:1367958. doi: 10.3389/fimmu.2024.1367958 (PMC11269137; doi:10.3389/fimmu.2024.1367958)
Supplement: Supplementary file 2 [file Table_1.docx]

Supplementary Figures

**Exploring Causal Correlations between Circulating Cytokines and Atopic Dermatitis: A Bidirectional Two-sample Mendelian Randomization Study**

**Zhenquan Xuan^1,2,3†^, Xuanyi Chen****^1,2,3†^, Weinan Zhou^1,2,3†^, Yihang Shen^1,2,3^, Zhe Sun^1,2,3^, Hui Zhang^1,2,3^****^*^, Zhirong Yao^1,2,3*^**

^1^Dermatology Center, Xinhua Hospital, Shanghai Jiaotong University School of Medicine, Shanghai 200082, China.

^2^Department of Dermatology, Xinhua Hospital, Shanghai Jiaotong University School of Medicine, Shanghai 200082, China.

^3^Institute of Dermatology, Shanghai Jiaotong University School of Medicine, Shanghai 200082, China.

^†^These authors contributed equally to this work and share first authorship.

**^*^Correspondence:**

Hui Zhang and Zhirong Yao

c_zhanghui@sina.com and yaozhirong@xinhuamed.com.cn


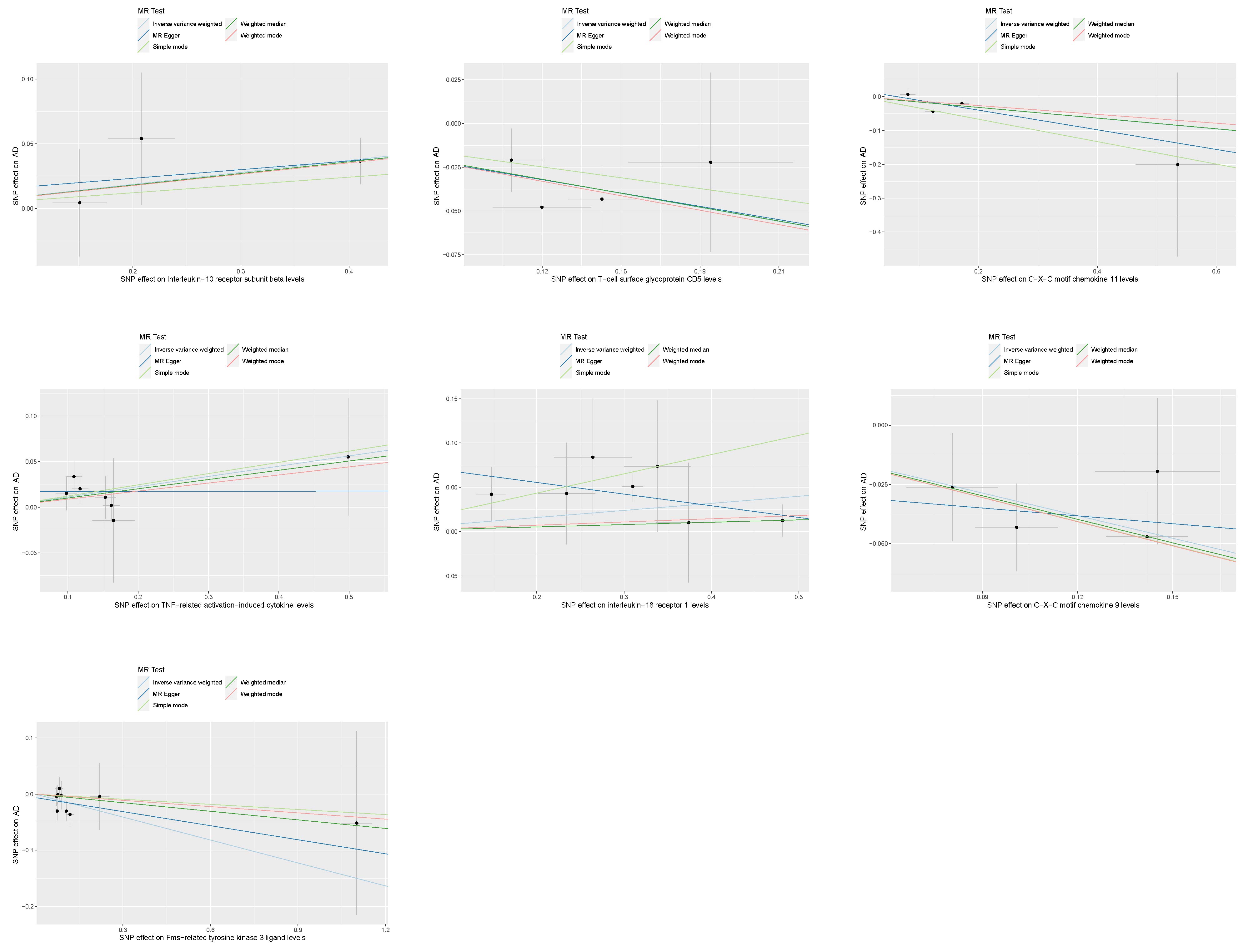


**Supplementary Figure S1.** Scatter plots of the impact of each SNP for each cytokine on atopic dermatitis (AD) (SNPs reaching P<5×10^-8^). The slope of the straight line indicates the magnitude of the causal association.


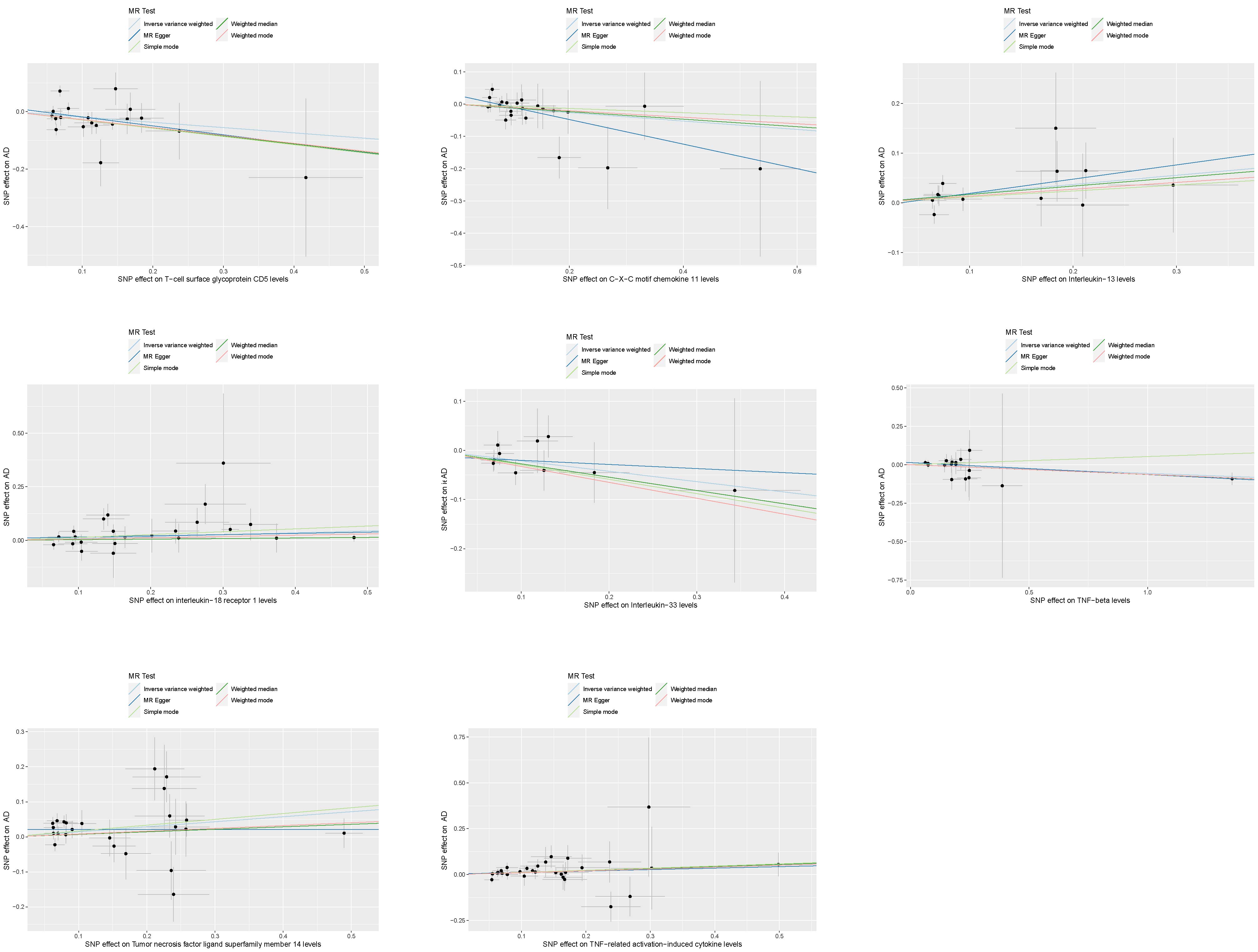


**Supplementary Figure S2.** Scatter plots of the impact of each SNP for each cytokine on atopic dermatitis (AD) (SNPs reaching P<5×10^-6^). The slope of the straight line indicates the magnitude of the causal association.


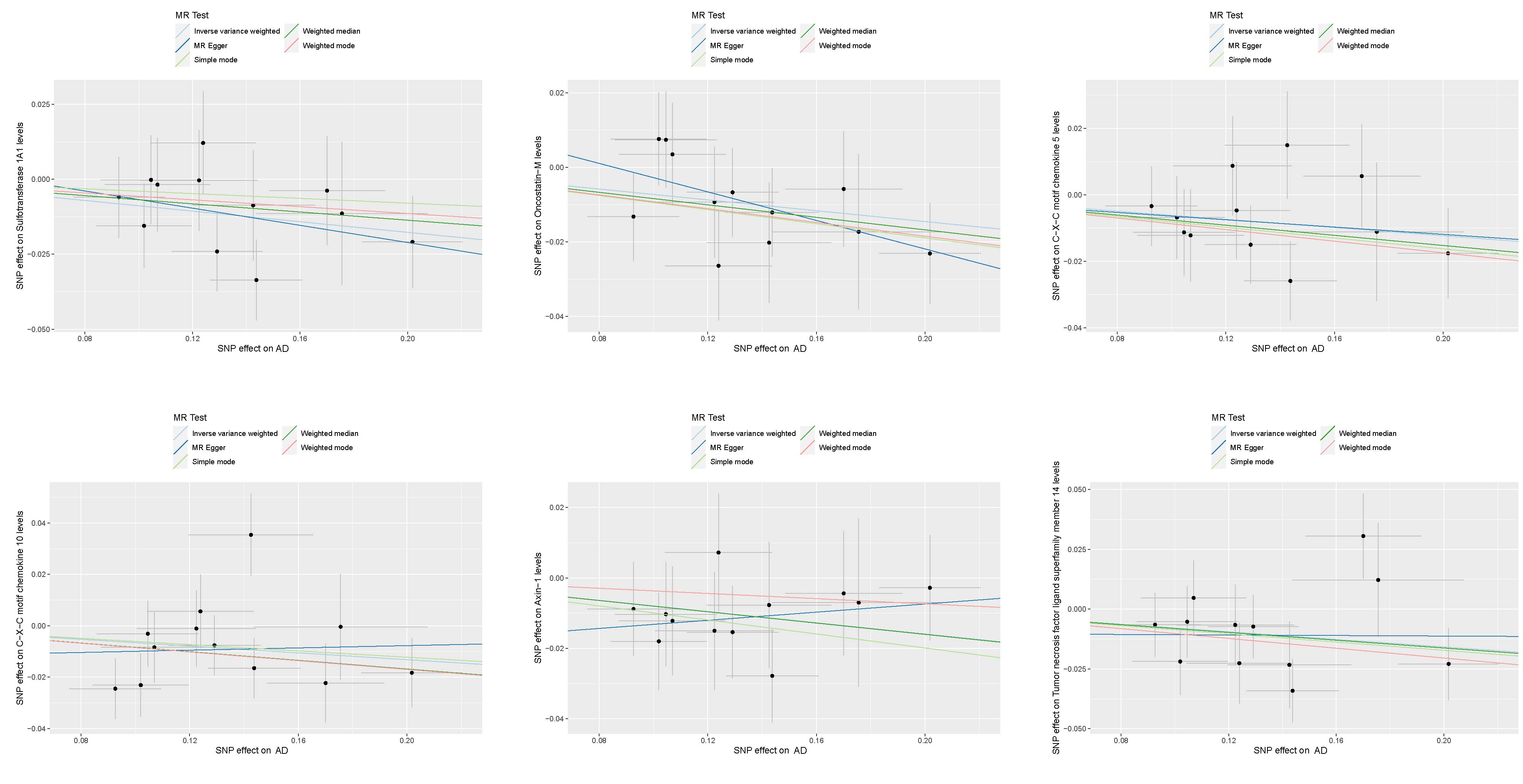


**Supplementary Figure S3.** Scatter plots of the impact of atopic dermatitis (AD) on six cytokines. The slope of the straight line indicates the magnitude of the causal association.





**Supplementary Figure S4.** Causal correlations of 8 cytokines with atopic dermatitis in the validation GWAS dataset (SNP associations reaching a significance level of P < 5×10^-6^). CXCL11, C-X-C motif chemokine 11; IL-13, interleukin-13; IL-18 R1, interleukin-18 receptor 1; IL-33, interleukin-33; TNF-β, TNF-beta; TNFSF14, Tumor necrosis factor ligand superfamily member 14; TRANCE, TNF-related activation-induced cytokine; CD5, T-cell surface glycoprotein CD5.


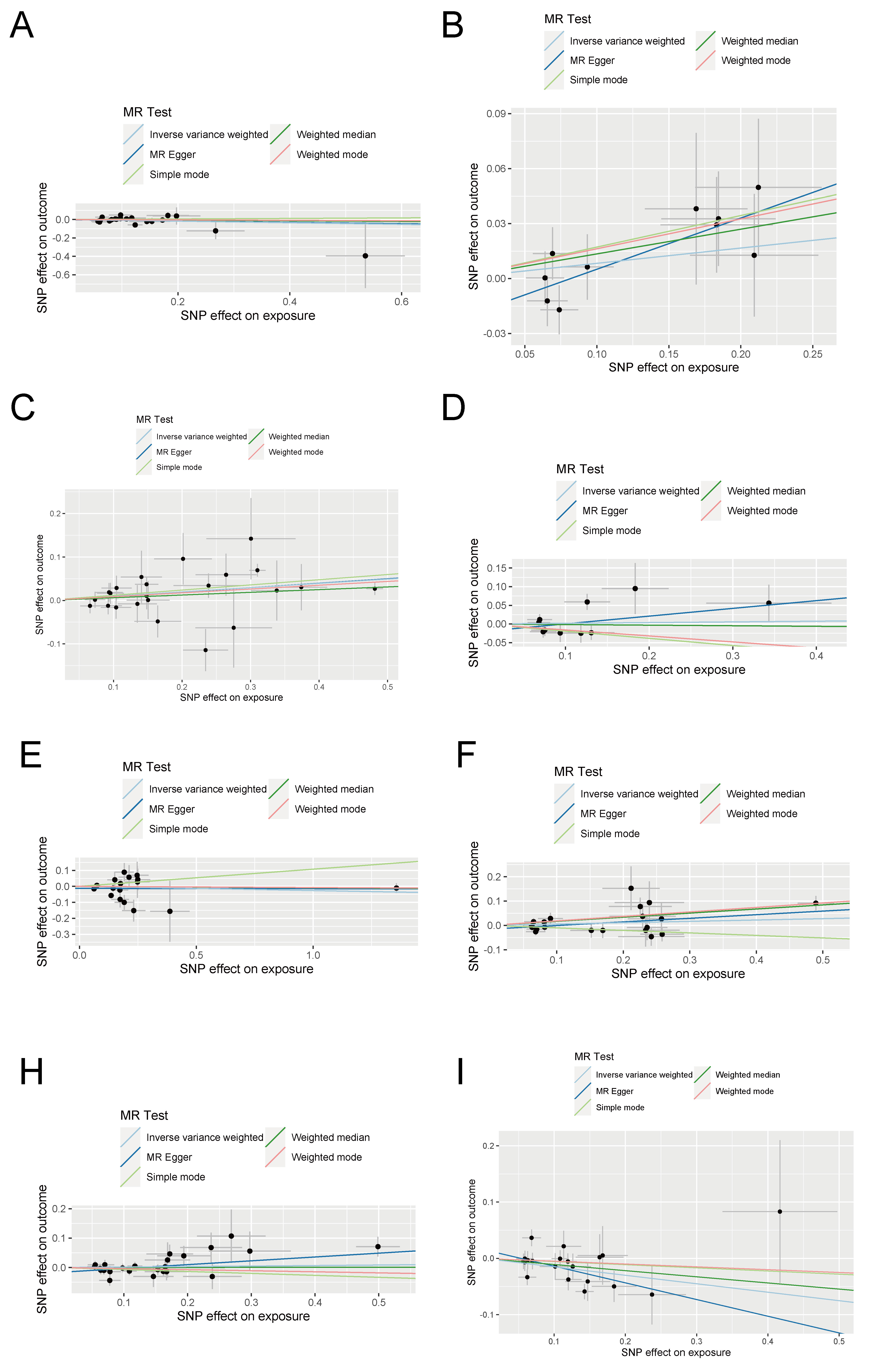


**Supplementary Figure S5.** The scatter plots of MR analyses used for the validation of the results in FinnGen GWAS data. Scatter plots presented the impact of each SNP for CXCL11 (A), IL-13 (B), IL-18R1(C), IL-33(D), TNF-β(E), TNFSF14(F), TRANCE(G) and CD5(H) on atopic dermatitis (AD) (SNPs reaching *P*<5×10^-6^). The slope of the straight line indicates the magnitude of the causal association.
